# Increasing accessibility to a large brain–computer interface dataset: Curation of physionet EEG motor movement/imagery dataset for decoding and classification

**DOI:** 10.1016/j.dib.2024.110181

**Published:** 2024-02-12

**Authors:** Zaid Shuqfa, Abderrahmane Lakas, Abdelkader Nasreddine Belkacem

**Affiliations:** a Connected Autonomous Intelligent Systems Lab, Department of Computer and Network Engineering, College of IT (CIT), United Arab Emirates University (UAEU), Al Ain City 15551, the United Arab Emirates; b Rabdan Academy, P.O. Box 114646, Abu Dhabi, the United Arab Emirates

**Keywords:** Brain–computer interface (BCI), Electroencephalography/electroencephalogram (EEG), Motor execution (ME), Motor imagery, Data curation, Dataset

## Abstract

A reliable motor imagery (MI) brain–computer interface (BCI) requires accurate decoding, which in turn requires model calibration using electroencephalography (EEG) signals from subjects executing or imagining the execution of movements. Although the PhysioNet EEG Motor Movement/Imagery Dataset is currently the largest EEG dataset in the literature, relatively few studies have used it to decode MI trials. In the present study, we curated and cleaned this dataset to store it in an accessible format that is convenient for quick exploitation, decoding, and classification using recent integrated development environments. We dropped six subjects owing to anomalies in EEG recordings and pre-possessed the rest, resulting in 103 subjects spanning four MI and four motor execution tasks. The annotations were coded to correspond to different tasks using numerical values. The resulting dataset is stored in both MATLAB structure and CSV files to ensure ease of access and organization. We believe that improving the accessibility of this dataset will help EEG-based MI-BCI decoding and classification, enabling more reliable real-life applications. The convenience and ease of access of this dataset may therefore lead to improvements in cross-subject classification and transfer learning.

Specifications Table

**Table:** 

Subject	Neuroscience (General)
Specific subject area	Brain-computer interface, Human-computer interaction, Motor imagery, Motor execution.
Data format	Raw, Filtered
Type of data	EEG Signal
Data collection	The BCI2000 system was used to record EEG from 109 subjects using 64 electrodes (international 10-10 system) at 160 Hz. The original dataset is available at (https://physionet.org/content/eegmmidb/1.0.0/)
Data source location	Institute: Wadsworth Center, New York State Department of Health, Albany, NY. The original dataset is hosted at https://physionet.org/content/eegmmidb/1.0.0/
Data accessibility	Repository name: Mendeley Data Data identification number: http://dx.doi.org/10.17632/dpmtgrn8d8 Direct URL to data: https://data.mendeley.com/datasets/dpmtgrn8d8[1]

## 1 Value of the Data

• This dataset is a curation of the largest publicly available MI and motor execution (ME) dataset. We reproduced the data in a clean, structured, and accessible format. A large number of subjects would benefit from multiple areas of MI-BCI, such as deep-learning MI-BCI, transfer and federated learning in BCI, BCI illiteracy/proficiency, and inter-/intra-subject decoding and control.
• The dataset comprises a relaxed class, four MI classes, and four ME classes, of which four are unilateral and four are bilateral. The variety of classes ensures versatility for the classification and decoding of experiments.
• The BCI research community will benefit from the dataset, as it is readily and publicly available for decoding, classification, and control.
• The dataset can be used to 1) decode MI trials in a binary classification setting: using the same task, either MI or ME; 2) decode MI trials in a multi-class classification setting, using two tasks of either MI or ME; 3) decode MI trials using bilateral or unilateral movements: selecting either fists vs. both feet or left fist vs. right fist; 4) experiment transfer learning using cross-subject tasks: calibrating on specific subjects and evaluating others employing relevant transfer-learning techniques; and 5) train deep-learning models using the large number of trial provisions.

## 2 Background

In motor-imagery (MI) brain-computer interfaces (BCIs), the subject controls the device spontaneously and asynchronously; in other words, subjects may freely generate control signals without external stimuli or cues from the system [[2], [3]]. In the MI paradigm, brain activity is generated by imagining the movement of a body part without the need to move muscles or use regular neural channels [[3], [4], [5]]. However, many prior electroencephalography (EEG)-based MI-BCI experiments had insufficient calibration and training data [6,7]. Because the process of acquiring sufficient signal recordings, preprocessing data and assigning relevant labels is highly resource intensive [8], a feasible solution is to exploit existing datasets.

To the best of our knowledge, the EEG Motor Movement/Imagery Dataset (EEGMMIDB) [9,10] is the largest EEG MI dataset available to the public in terms of the number of subjects [3], offering more than 1500 EEG recordings of 109 subjects performing eight tasks (four MI and four ME tasks). Despite its size and versatility, EEGMMIDB has received relatively little research attention since its initial publication in 2009 [[11], [12], [13]]. By investigating other datasets, we found that this dataset was not well-prepared for direct use by researchers, as the recordings are stored in a primitive format – namely the European data format “plus” (EDF+) [10,14] – which can only be accessed using specific toolboxes and libraries. Furthermore, the dataset includes incomplete recordings, and the coding of annotations is difficult when considering different task files. In contrast, more popular datasets can be accessed using widespread integrated development environments (IDEs) that are cleaned and organized for direct use. We believe that converting EEGMMIDB to an accessible, structured, clean, and validated form may enhance its reusability and exposure, thereby contributing to MI-BCI research. Our endeavor to produce such a dataset includes the following considerations:

• The resulting dataset adds significant value to the original data in terms of cleaning, structuring, validation, and accessibility.
• A scientific protocol for data collection and curation is well-prescribed for reproducibility and validation.
• The resulting dataset is publicly available for the benefit of BCIs and the computational neuroscience research community.

## 3 Data Description

**Fig. 1:**
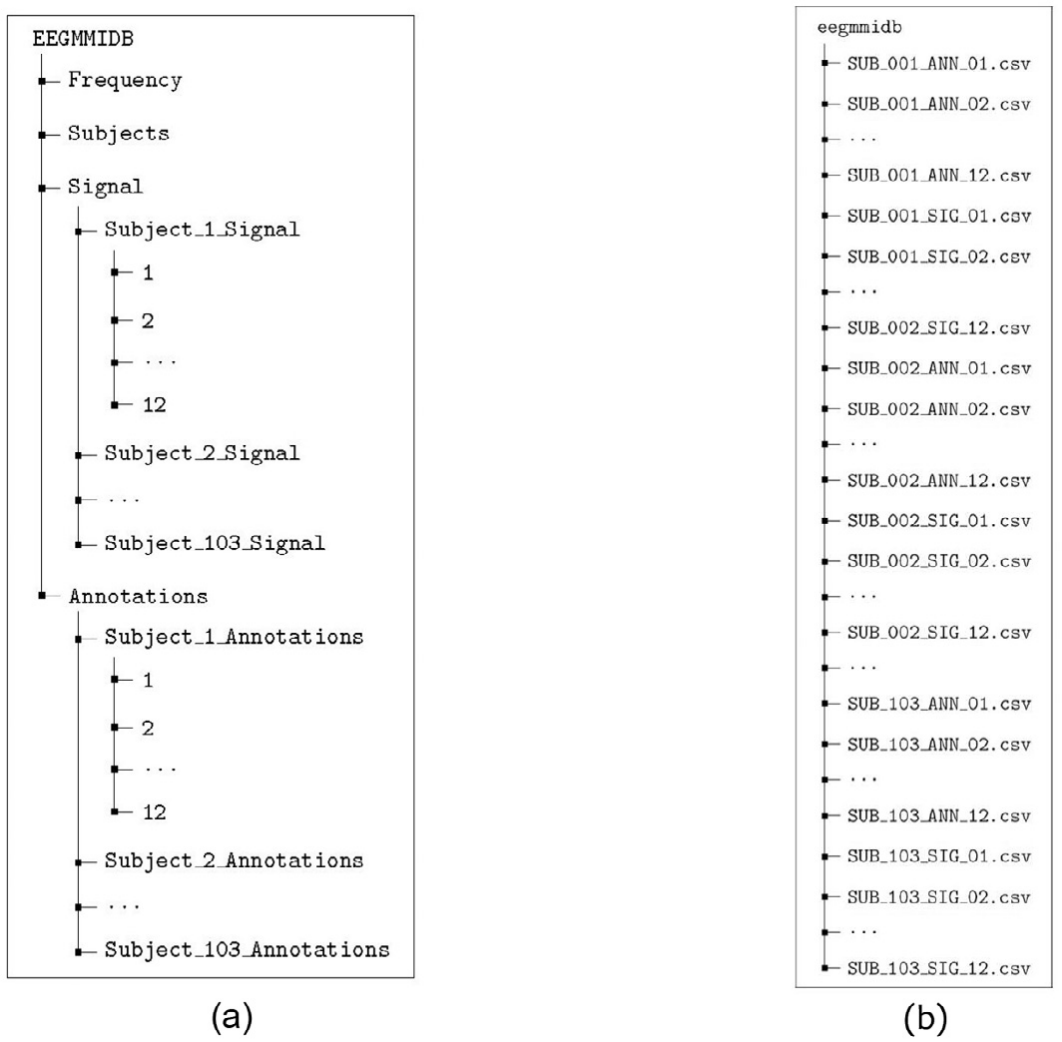
Dataset content. (a) EEGMMIDB MATLAB structure and (b) eegmmidb folder containing csv files.

The dataset encompasses EEG recordings of 103 subjects who performed different ME and MI tasks. Data were organized in a MATLAB structure, EEGMMIDB, as the tree depicted in Fig. 1 (a), and stored in comma-separated value (CSV) files. Fig. 1 (b) illustrates the organization of the folder (eegmmidb) containing the CSV files. In EEGMMIDB, Frequency is a scalar representing the frequency of the recordings, set to 160 Hz. Subjects are numerically represented by a matrix, Subjects, where the first row represents the original subjects while the second row represents all remaining subjects following the exclusion of inconsistent recordings. For additional details pertaining to discarded records, please refer to (EXPERIMENTAL DESIGN, MATERIALS AND METHODS). Signal and Annotations are structures containing data records.

The structure Signal consists of 103 cell arrays identified by the subject number, e.g., Subject_1_Signal. Each cell array contains 12 matrices of size (), holding the readings of 64 electrodes over a two-minute run at a 160 Hz. sampling rate. For details on the runs, please refer to Tables 1 and 2. Similarly, the Annotations structure contains 103 cell arrays identified by subject number, e.g., Subject_46_Annotations. Each cell array contains 12 matrices of size (), each row of which corresponds to a trial in the respective signal matrix. Each of the 30 rows contains the following fields: (1) trial label as in Table 2; (2) trial length in seconds; (3) trial length in data samples; and (4) and (5) trial onset and end, respectively, as indices of the corresponding signal matrix (see Fig. 1). It should be noted that array indexing in MATLAB begins at 1 rather than 0.

**Table 1:** Dataset parameters.

Parameter	Value
Subjects	103
Runs files per subject	12
Run length	2 min.
Tasks	4
Runs per task	3
Trials per run	30
Electrodes	64
Sampling rate (Hz.)	160

**Table 2:** Dataset run specifications.

Task	Description of the task	Mode	Type	Original dataset				Curated dataset			
				Run file number	Annotations			Signal and annotation reference	Class		
					T0	T1	T2		T0	T1	T2
Task 1	Open and close right or left fist	Motor Execution	Unilateral	3, 7, 11	Relax	Left fist	Right fist	1, 5, 9	1	2	3
Task 2	Imagine opening and closing right and left fists	Motor Imagery	Unilateral	4, 8, 12	Relax	Left fist	Right fist	2, 6, 10	4	5	6
Task 3	Open and close both fists and feet	Motor Execution	Bilateral	5, 9, 13	Relax	Both fists	Both feet	3, 7, 11	7	8	9
Task 4	Imagine opening and closing both fists and feet	Motor Imagery	Bilateral	6, 10, 14	Relax	Both fists	Both feet	4, 8, 12	10	11	12

The CSV files are stored in the eegmmidb folder, which is compressed into a WinRAR archive. All CSV files follow a unified naming convention to facilitate dynamic access. The signal files are named SUB_[sss]_SIG_[rr].csv, whereas the annotation files are saved as SUB_[sss]_ANN_[rr].csv, where [sss] represents the subject number from 001 to 103 and[rr] represents the run number from 01 to 12. Refer to column “Signal and annotation reference” in Table 2 for information pertaining to the task and classes associated with every run. Of the 2,472 total CSV files, half represent signal matrices while the other half store annotations.

To access the data variables, the binary MATLAB file EEGMMIDB_Curated.mat (mat file version 7.3) from http://dx.doi.org/10.17632/dpmtgrn8d8 can be loaded into MATLAB by double-clicking. Alternately, it can be loaded by keying the following command in MATLAB's command window: load '[directory]\EEGMMIDB_Curated' where [directory] corresponds to the directory on the machine.

To access the data in CSV format, unzip the archive and open the files in an appropriate IDE.

This dataset is a versatile collection of MI and ME recordings, which can be used to

• Decode MI trials in a binary classification setting using either MI or ME tasks.
• Decode MI trials in a multiclass classification setting using pairs of either MI or ME tasks.
• Decode motor imagery trials using bilateral or unilateral movements: selecting either fists vs. both feet or left fist vs. right fist.
• Conduct experimental transfer learning using cross-subject tasks (calibrate on specific subjects and evaluate others).
• train deep-learning models using the considerable quantity of data.

## 4 Experimental Design, Materials and Methods

The original EEGMMIDB data were recorded at 160 Hz using BCI2000, a general-purpose BCI system [15] on the international 10-10 system excluding Nz, F9, F10, FT9, FT10, A1, A2, TP9, TP10, P9, and P10, resulting in 64 electrodes (channels).

**Fig. 2:**
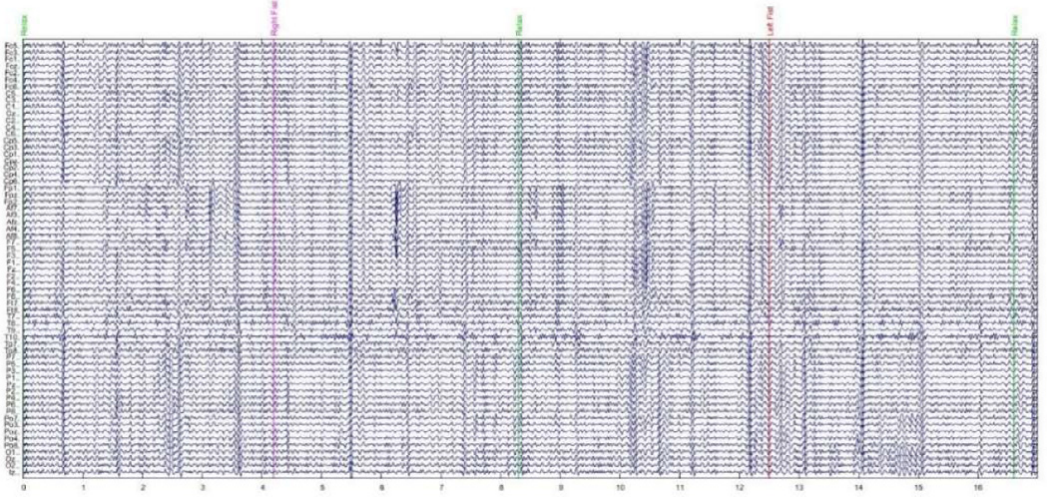
EEG recordings from Subject 1. First 17 s of run file number 4 (imagine opening and closing right vs. left fists). Band-Pass filtered at 8–30 Hz, where the *x*- and *y*-axes represent time and the 64 electrodes, respectively.

Each subject produced 14 run files: two baseline runs and 12 task runs (six each for MI and ME). For more details on the tasks performed, please refer to Table 2. Each run file comprised 15 MI or ME trials preceded by a relaxation period (Fig. 2). All trials and relaxation periods were of four-second length, resulting in a two-minute run file. The annotations for the trials – T0, T1, and T2 – differed with respect to task (see Table 2).

To make the dataset available for exploratory analysis and curation, the files were downloaded using the WFDB Toolbox for MATLAB and Octave [10,16,17]. Exploratory data analysis, cleansing, organization, and transformation were performed using MATLAB (R2021a) Update 3 [18] on a Windows 10 64-bit home edition. First, we excluded subjects with signal records that were inconsistent with the experimental description in [9] and inconsistent with other files. Accordingly, we assigned them new sequential IDs (1–103). Next, we dropped the baseline runs while retaining the ME and MI runs and renumerated the reference trials from 1 to 12 (Table 2). We then transformed the annotations for ease of reference and signal epoching depending on the desired application. Subsequently, the data were saved in accessible common EDIs formats: MATLAB structure and CSV files. The curated dataset was uploaded to an open-access repository, namely Mendeley Data.

The data extraction generated two matrices per run: a signal matrix of size (), where is the number of data points and is the number of channels (electrodes), and an annotation array of size , where is the number of trials in the run (ideally, 30 trials). The signal matrix contains electrode readings over time at 160 Hz. whereas the annotation array contains text annotations – e.g., T0 duration: 4.2 – corresponding to certain data elements (see Fig. 4).

**Fig. 3:**
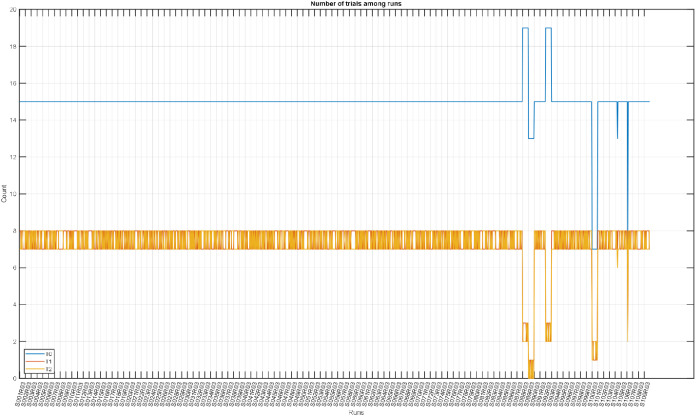
Count of trials in each run. Each point represents the number of trials in a run file. The blue curve shows the number of T0 trials while the orange and yellow curves represent T1 and T2, respectively. In most runs, T0 maintained an expected 15 occurrences, while T1 and T2 appeared either 7 or 8 times interchangeably. Some anomalies appeared when moving toward later subjects where the pattern was insufficiently clear.

To interpret the data and their characteristics, as well as identify anomalies, an exploratory data analysis was conducted, revealing anomalies stemming from missing trials (i.e., those trials with a length of 0). Another set of files was shorter than expected. We found that some run files were inconsistent with their data descriptions owing to missing trials. Table 4 and Fig. 5 show the lengths of all runs and their trials, where shorter-than-expected runs correspond to points where the blue curve falls below 120 s. We expected to observe approximately 60 s. of relaxation trials (T0) and approximately 28–32 s. of each T1 and T2 trial. Similarly, the run files were expected to have 7 to 8 MI or ME trials with 15 relaxation trials. Fig. 3 shows the number of trials for each run file, and Table 4 lists the anomalies of the discarded files. Participants with inconsistent lengths or numbers of trials were excluded. Table 4 lists the discarded subjects, whereas Table 3 lists the expected values found in most of the run files. When cleaning the data, we discarded six subjects and retained the following 103 subjects: S001-S087, S090-S091, S093-S099, S101-S103, S105, and S107-S109. These subjects were then numbered from 1 to 103, and the original sequence was retained. An annotation matrix was used to specify the onset and end of each trial. The 65th column of the signal matrix was found to be unnecessary and was dropped because the data were obtained from the annotation arrays.

**Table 3:** Typical subject parameters.

Number of trials in the runs	Length of trials in the run	Length of the runs
15relax 7−8MEandMI	≅4.1±0.2s.	≅123.5s.

**Table 4:** Reasons for discarding subjects.

#	Discarded subject	Number of trials in the runs	Length of trials in the run	Length of the runs
1	S088	19relax 2−3MEandMI	1.375−5.125s.	51.75s.
2	S089	13relax 0−1MEandMI	0−4.1s.	56.8s.
3	S092	19relax 2−3MEandMI	1.375−5.125s.	51.75s.
4	S100	7relax 1−2MEandMI	5.125s.	51.25s.
5	S104	13relax 6MEandMI	2.975−4.1s.	53.30s.
6	S106	5relax 2MEandMI	3.1−4.2s.	36.30s.

**Fig. 4:**
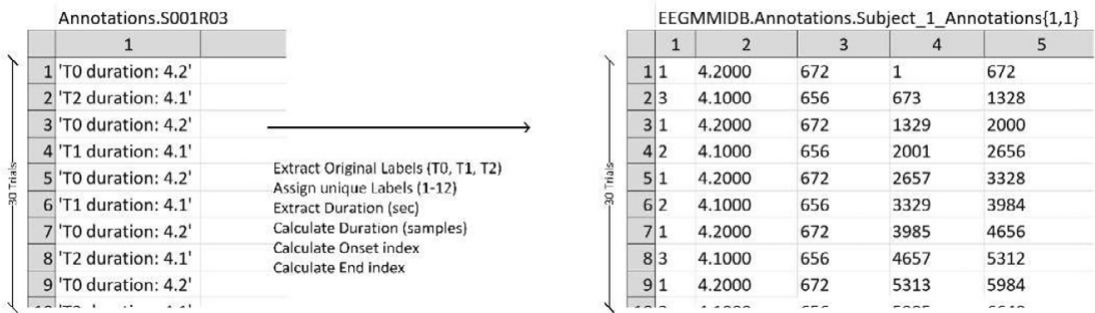
Transforming Annotations arrays.

The annotations in the annotation matrix were used to classify trials with labels T0, T1, and T2 from the different runs. Depending on the task, the annotation was assigned a number, as shown in Table 2 to facilitate classification between classes from different trials. Fig. 4 shows the data transformation toward structured annotations. Initially, annotations were extracted as T0, T1, and T2. Next, numeric labels from 1 to 12 were assigned as described in Table 2. The trial duration was then calculated in terms of data samples rather than seconds. Accordingly, the movement onset was designated by index. Finally, the end of each trial was recorded.

**Fig. 5:**
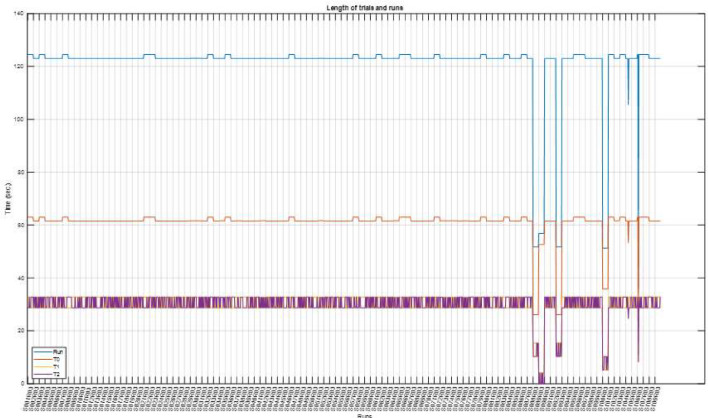
Lengths of runs and trials: Total length of each run in seconds in comparison to the total length of annotations in each run. The x-axis represents the runs of the subjects. The tick marks show subjects (from Run 3 to Run 14) where every data point on the chart represents one run.

## Limitations

Not applicable.

## Ethics Statement

The authors have read and followed the ethical requirements for publication in Data in Brief. They confirm that the current work does not involve human subjects, animal experiments, or any data collected from social media platforms. The data in this manuscript is secondary data and we did not need permission to use the primary data sources.

## CRediT authorship contribution statement

**Zaid Shuqfa:** Conceptualization, Methodology, Software, Validation, Investigation, Data curation, Writing – original draft, Visualization. **Abderrahmane Lakas:** Conceptualization, Validation, Resources, Writing – review & editing, Supervision, Project administration, Funding acquisition. **Abdelkader Nasreddine Belkacem:** Conceptualization, Validation, Writing – review & editing, Supervision.

## Data Availability

Physionet EEGMMIDB in MATLAB structure to leverage accessibility and exploitation (Original data) (Mendeley Data) EEG Motor Movement/Imagery Dataset (Reference data) (Website)
